# Reversing immunosenescence for prevention of COVID-19

**DOI:** 10.18632/aging.103636

**Published:** 2020-06-28

**Authors:** Robert T. Brooke, Gregory M. Fahy

**Affiliations:** 1 Intervene Immune, Inc., Torrance, CA 90502, USA

**Keywords:** epigenetic aging, thymus, thymic hormones, immune senescence, COVID-19

Age is the strongest predictor of the severity and lethality of COVID-19 [1]. But is age - or at least biological age - a modifiable risk factor? Our recently published TRIIM trial [2] demonstrated that in healthy older adults it is possible to not only regenerate the thymus and reverse age-related immunological changes but also to reverse epigenetic aging - the most robust indicator of biological age available today [3]. These observations introduce new possibilities for preventive medicine in the elderly, and the nature of SARS-CoV-2 infection suggests new possibilities may be necessary.

The primary reason advanced age increases susceptibility to COVID-19, and all other infectious diseases, is an age-related decline of immune competence, or immunosenescence [1,2]. Two hallmarks of this process are the well-known loss with age of naïve T cell generation and T cell diversity [1,2], which provide the source of the resilience and versatility of the cellular component of the adaptive immune system and are also important for humoral immunity - the mounting of robust antibody responses. This problem, and potentially a good deal of aging more generally, is made inevitable by the involution of the thymus in early life [2]. The thymus produces naïve T cells that can help recognize and clear infectious agents, including viruses like SARS-CoV-2, from the body, through both helper CD4 T cells and cytotoxic CD8 T lymphocytes. Age-related loss of thymic T cell output leads eventually to a reduced capacity to mount robust adaptive immune responses to novel antigens in later life [2].

Virus-specific T cell responses have been shown to be present in 100% of individuals who recover from COVID-19 [4]. In the elderly, generation of a similar response is presumably more difficult due to the tendency of COVID-19 to induce a profound depression of circulating total T cells, CD8^+^ T cells, and also NK cells and an increase in functionally exhausted T cells [5] in the presence of pre-existing deficits in T cell receptor repertoire. In principle, these deficits may be corrected by thymus regeneration, as our recent trial demonstrated that regeneration of the thymus was accompanied by increases in naïve T cells and recent thymic emigrants and by decreases in the number of exhausted CD8 T cells in normal aging men [2]. Interestingly, the first demonstrations of thymus regeneration in humans were inspired by the T cell depleting effects of the HIV virus, rather reminiscent of the effect of SARS-CoV-2, and those demonstrations were successful in improving T cell levels despite ongoing viral infection [6]. T cell repletion by thymus regeneration could also be long-lasting. In the case of SARS-CoV-1 infection, which is closely related, virus-specific CD8 T cells have been shown to be able to persist for at least 11 years post-infection [7].

Thymic involution also brings with it reduced production of thymic hormones such as thymosin alpha-1. Recently, thymosin alpha-1, by itself, was found to reduce COVID-19-associated mortality [8], providing additional evidence that thymus regeneration may help to prevent or moderate this disease. The potential for thymus regeneration to help protect older individuals from COVID-19 and immune system aging more generally will soon be further investigated through our expanded TRIIM-X clinical trial.

As a preventive medicine measure, the TRIIM treatment is unique in that it addresses the reversal of both epigenetic aging and immunosenescence, and does this using a combination of FDA-approved drugs that are already available today [2]. It involves use of growth hormone as a thymotrophic agent, which has been well established in both preclinical and clinical studies to induce the production of new naïve T cells and to enhance immune system function, and complementary agents that block side effects and may have independent benefits of their own.

Today’s medicine, dramatic public health measures, new vaccines and treatments, and perhaps natural attenuation will presumably beat back COVID-19 later this year, but a more permanent and fundamental solution is needed. Immunosenescence, more than anything else, is what makes us susceptible to COVID-19, influenza, pneumonia, countless other infectious diseases, and very likely also many of the risks of cancer and even cardiovascular disease through increased inflammation. Unless we address immunosenescence in a powerful way, it will continue to plague us long after we’ve contained this particular pandemic. Fortunately, humanity has more treatments and diagnostics in its arsenal than ever before. We also have new digital health technologies that can enable more efficient, larger, and more definitive clinical trials - better science - than ever before. Combining these new technologies with treatments to reverse immunosenescence could enable us to protect our elderly and provide health and economic benefit to the broader community for decades to come.
